# Development of severe colitis is associated with lung inflammation and pathology

**DOI:** 10.3389/fimmu.2023.1125260

**Published:** 2023-03-31

**Authors:** April L. Raftery, Caitlin A. O’Brien, Nicola L. Harris, Evelyn Tsantikos, Margaret L. Hibbs

**Affiliations:** Department of Immunology and Pathology, Central Clinical School, Monash University, Melbourne, VIC, Australia

**Keywords:** gut-lung axis, colitis, inflammatory bowel disease, lung inflammation, γδ T cells

## Abstract

Inflammatory bowel diseases (IBD) such as Crohn’s disease and ulcerative colitis are chronic relapsing diseases that affect the gastrointestinal tract, most commonly the colon. A link between the gut and the lung is suggested since patients with IBD have an increased susceptibility for chronic inflammatory lung disease. Furthermore, in the absence of overt lung disease, IBD patients have worsened lung function and more leukocytes in sputum than healthy individuals, highlighting a conduit between the gut and lung in disease. To study the gut-lung axis in the context of IBD, we used TCRδ^-/-^ mice, which are highly susceptible to dextran sulfate sodium (DSS) due to the importance of γδ T cells in maintenance of barrier integrity. After induction of experimental colitis using DSS, the lungs of TCRδ^-/-^ mice exhibited signs of inflammation and mild emphysema, which was not observed in DSS-treated C57BL/6 mice. Damage to the lung tissue was accompanied by a large expansion of neutrophils in the lung parenchyma and an increase in alveolar macrophages in the lung wash. Gene expression analyses showed a significant increase in *Csf3*, *Cxcl2*, *Tnfa*, and *Il17a* in lung tissue in keeping with neutrophil infiltration. Expression of genes encoding reactive oxygen species enzymes and elastolytic enzymes were enhanced in the lungs of both C57BL/6 and TCRδ^-/-^ mice with colitis. Similarly, surfactant gene expression was also enhanced, which may represent a protective mechanism. These data demonstrate that severe colitis in a susceptible genetic background is sufficient to induce lung inflammation and tissue damage, providing the research community with an important tool for the development of novel therapeutics aimed at reducing co-morbidities in IBD patients.

## 1 Introduction

Inflammatory bowel diseases (IBD) are chronic, relapsing diseases that affect the gastrointestinal tract and can be broadly divided into Crohn’s disease and ulcerative colitis. IBD is characterized by immune cell infiltration, intestinal barrier hyperpermeability, and dysbiosis of the intestinal microbiota (1). Epidemiological studies have revealed that IBD patients are more likely than the general population to develop chronic inflammatory lung diseases such as chronic obstructive pulmonary disease (COPD) and asthma (2–5). Furthermore, IBD patients without overt lung disease have reduced lung function and increased inflammatory cells in their sputum compared to healthy individuals (6). Conversely, studies have shown that COPD patients exhibit functional alterations to the gut when at rest, such as intestinal barrier hyperpermeability, which is worsened with general daily activity and acute exacerbations (7, 8). Together, these studies suggest that chronic inflammation in either the gut or the lungs can impact upon the other.

The gut-lung axis is a widely accepted bi-directional interaction that occurs between the two mucosal organs. To date, a wealth of studies in this area have investigated the importance of gut microbes and their metabolites on lung diseases (9, 10). However, less is known about the direct impact of inflammatory crosstalk irrespective of the microbiota. Given the correlation between IBD and COPD, we set out to investigate whether the presence of gut pathology altered lung inflammation *via* this axis. While it is unclear why IBD patients are predisposed to pulmonary inflammation and lung disease, it is possible that there is spillover of intestinal inflammation into the systemic circulation that can influence distal sites such as the lung due to inherent similarities in these organs, which have both derived embryonically from the primitive foregut (11) and include mucosal surfaces (12). This immunological component of the gut-lung axis in the form of circulating cytokines and leukocytes may be an important mechanism that underpins the development of comorbidities associated with the lung. A previous study examining the lung following induction of colitis in mice with dextran sulfate sodium (DSS) found that intestinal inflammation induced infiltration of neutrophils and monocytes to the lungs, a mechanism dependent on microbial spill over from the gut to the lungs; however, this did not provoke lung damage or pathology (13).

γδ T cells have been extensively studied in the context of IBD, both in animal models and patients, as playing a protective role through the production of interleukin (IL)-17A, which is important in regulating intestinal barrier permeability (14). This is highlighted by the severe outcomes observed in IBD patients in clinical trials with the anti-IL-17A drug secukinumab, which resulted in intestinal barrier hyperpermeability and exacerbation of disease (15). The importance of γδ T cells and IL-17A in IBD pathogenesis makes TCRδ^-/-^ mice an ideal model for studying the gut-lung axis and lung inflammation in the context of IBD. The impact of TCRδ-deficiency on the colon has been extensively studied using the DSS-induced colitis model. TCRδ^-/-^ mice exhibit increased intestinal barrier permeability following DSS treatment as a result of impaired intestinal epithelial cell proliferation due to reduced IL-17A and keratinocyte growth factor (KGF) expression in the colon (14, 16, 17). Furthermore, TCRδ^-/-^ mice are more susceptible to inflammation following lung infections such as *Mycobacterium tuberculosis*, *Streptococcus pneumoniae*, and influenza (18–20), which is relevant since a proposed mechanism of lung inflammation in association with IBD is bacteremia as well as gastrointestinal and respiratory tract dysbiosis (10, 13). Thus, being susceptible to both intestinal and respiratory inflammation, the TCRδ^-/-^ model is ideal for assessing the resulting inflammation in the lungs following induction of colitis.

Here in this study, we utilized DSS-treated TCRδ^-/-^ mice as a model of more pronounced colitis (16, 17, 21, 22) to determine whether severe intestinal inflammation can elicit lung disease and damage following leukocyte recruitment. We demonstrate that DSS-induced colitis is sufficient to drive acute lung inflammation with resulting histological changes. Lung inflammation is primarily associated with neutrophil infiltration and activation, which promotes lung pathology.

## 2 Materials and methods

### 2.1 Animals

Adult TCRδ^-/-^ mice on a C57BL/6 background (23) were used for these studies and age-matched C57BL/6 mice were used as controls. Mice were bred at the Monash Animal Research Precinct, Clayton, Australia then shipped by Jetpets (Melbourne, Australia) to the Precinct Animal Centre at the Alfred Research Alliance, Melbourne, Australia 2 weeks prior to commencement of DSS treatment, both of which facilities are specific pathogen free. Mice of different treatments and genotypes were housed separately in boxes of 4 - 5 mice, all boxes were kept in the same section of the rack. Mice were housed on autoclaved dust free Aspen Chips (Biological Associates), fed a standard irradiated (Steritech, Australia) rat and mouse diet (Specialty Feeds, Australia), and kept at 19 - 23°C with a relative humidity of 55 ± 15. Enrichment was provided in the form of autoclaved cardboard boxes, cardboard rolls, egg cartons, or popsicle sticks, which were alternated weekly with cage cleans. Experiments were performed according to National Health and Medical Research Council of Australia (NHMRC) guidelines and approved by the Animal Ethics Committee of the Alfred Research Alliance, Melbourne (project numbers E1830/2018/M and E8248/2021/M).

### 2.2 Dextran sulfate sodium (DSS) treatment

Adult male mice of 12 weeks of age were provided 2.5% (w/v) colitis grade (36,000 – 50,000 MW) DSS salt (MP Biomedicals) in autoclaved drinking water ad libitum for 7 days, followed by drinking water for 1 day prior to assessment. Control male mice were given autoclaved drinking water. Male mice were used for this study since female mice are partially protected from DSS-induced colitis (24). Mice were monitored daily for weight loss, diarrhea, rectal bleeding, and general body condition and were euthanized if they lost more than 20% of starting weight or showed severe signs of colitis. Mice were excluded from analysis if euthanized more than one day prior to designated endpoint. Weight data was excluded from analysis if not collected on the same set of scales as previous data to maintain consistency.

### 2.3 Bronchoalveolar lavage

Bronchoalveolar lavage (BAL) was performed on lungs from terminally anesthetized mice by washing with 400 μL of ice-cold phosphate-buffered saline (PBS) followed by a further three washes of 300 μL. Cells were pelleted, BAL fluid (BALF) was taken from the first lavage, and then all lavages were pooled. Cell counts were determined by hemocytometer and are presented as total cells of recovered volume which ranged between 1.03 mL and 1.21 mL. Cells were centrifuged onto glass slides and stained using Hemacolor® (Sigma-Aldrich, Darmstadt, Germany).

### 2.4 Cytokine analysis of BALF and plasma

Cytokine and chemokine concentration in BALF and plasma were analyzed using two multiplex kits; Bio-Plex Pro™ Mouse Cytokine Grp 1 Panel 23-Plex for G-CSF, GM-CSF, IFN-γ, IL-1α, IL-1β, IL-3, IL-4, IL-6, IL-9, IL-10, IL-12p40, IL-12p70, IL-13, IL-17A, KC (CXCL1), RANTES (CCL5), and TNF-α (Bio-Rad, Hercules, CA, USA), and Bio-Plex Pro™ Th17 Cytokine 10-Plex Panel for CD40L, IL-17F, IL-22, IL-23, IL-25/IL-17E, IL-27, IL-31, and MIP-3α (CCL20) (Bio-Rad, Hercules, CA, USA). Analysis was performed using the Bio-Plex® 200.

### 2.5 Histology

Colons were washed with ice-cold PBS, rolled using the Swiss roll technique and fixed in 10% neutral-buffered formalin (10% NBF). After BAL wash was performed lungs were inflation-fixed in 10% NBF as previously described (25). Tissues were paraffin-embedded, sections were cut to a thickness of 4 μm, and stained with hematoxylin and eosin (H&E). Slides were scanned (Aperio AT2) and analyzed using the Aperio ImageScope digital slide viewer (v12.4.0.5043). Colitis was graded as previously described (13). Alveolar airspace size was quantified using the mean linear intercept (MLI) method (26). Briefly, 20 equal length lines were drawn over images of H&E-stained lung cross-sections, then the number of intercepts with alveolar walls were counted. MLI was the result of dividing the line length with the average intercepts per line.

### 2.6 Single cell preparation

After being lavaged, lungs, which were not perfused, were digested with Liberase (Merck) and DNase I (Sigma-Aldrich) using the gentleMACS Octo Dissociator with Heaters (Miltenyi Biotec) and the 37C_m_LDK_1 preinstalled setting before filtering with a 70 μM cell filter. Single-cell suspensions of the intraepithelial lymphocyte (IEL) population of the colon were prepared as previously described (27). Briefly, the intestines were flushed with ice-cold PBS, before being cut into ~1 cm long pieces, and washed. The intestinal pieces were then finely cut, placed in EDTA solution (HBSS, 0.5% FCS, 15mM HEPES, 5mM EDTA), and shaken at 100 rpm for 40 minutes at 37°C before being filtered and washed.

### 2.7 Flow cytometry

Single cell suspensions of lung, colon IELs, bone marrow, spleen, and BAL cells had Fc receptors were blocked with anti-FcγRII/III (2.4G2, in-house), were stained with fluorophore-conjugated monoclonal antibodies and analyzed by flow cytometry on an LSR-Fortessa X-20 (BD Biosciences). The following reagents were used: BUV395-anti-CD45 (30-F11, BD Biosciences), APCe780-anti-CD11b (M1/70, eBioscience), Alexa Fluor 700-anti-CD11c (N418, eBioscience), PerCP Cy5.5-anti-Gr-1, BV421-anti-Siglec F (E50-2440, BD Biosciences), FITC-anti-CD3 (145-2C11, BD Bioscience), PerCP Cy5.5-anti-CD8 (53-6.7, BD Biosciences), BV510-anti-CD4 (RM4-5, Biolegend), PE-anti-γδ TCR (GL3, Biolegend), APCe780-anti-CD44 (IM7, eBioscience), PECy7-anti-CD62L (MEL-14, Biolegend), FITC-anti-CD71 (C2, BD Bioscience), PE-anti-Ter119 (TER-119, BD Bioscience), PE-anti-Sca-1 (D7, BD Bioscience), FITC-anti-cKit (2B8, BD Bioscience), biotinylated anti-CD16/32 (2.4G2, BB Bioscience), BUV737-anti-F4/80 (T45-2342, BD Bioscience), APC-anti-CD11b (M1/70, BD Bioscience), APC-anti-B220 (RA3-6B2, BD Bioscience), APC-anti-Gr-1 (RB6.8C5, BD Bioscience), APC-anti-CD11c (HL3, BD Bioscience), APC-anti-CD4 (RM4-5, BD Bioscience), APC-anti-CD8 (53-6.7, BD Bioscience), APC-anti-CD5 (53-7.3, BD Bioscience), APC-anti-I-Ab MHC II (AF6-120.1, BD Bioscience), BV711 streptavidin (BD Bioscience). Fluorogold viability dye was used to gate on viable cells. FlowJo software (Windows v10, FlowJo LLC) was used to analyze acquired data. Absolute numbers of cells were calculated from cell counts acquired on a Coulter counter (Beckman Coulter) and proportions determined by flow cytometry. Populations were defined as indicated in **Supplementary Figure 1**. Expression of activation and phenotypic markers (Gr-1, CD11b, CD45, CD62L, Ly6G, Siglec F) were determined by normalizing the geometric mean fluorescence intensity (gMFI) by dividing the value of each sample by the mean value of the C57BL/6 H_2_O group within each experiment, allowing pooling of data from multiple experiments.

### 2.8 Quantitative reverse transcription polymerase chain reaction (qRT-PCR)

RNA was isolated from the middle right lung lobe using trizol and then converted to cDNA according to the manufacturer’s protocol (FireScript, Solis BioDyne). cDNA was used for qRT-PCR analysis using SYBR green PCR Master Mix (Applied Biosystems). Genes of interest were assessed using SYBR-green primers (**Table 1**). Eukaryotic *18S* rRNA was used as the control for all samples. CT values were calculated using automatic threshold analysis (QuantStudio Software), with results of triplicates averaged. The relative expression of each target gene was calculated by the 2^-∆∆CT^ method and was normalized to *18s* expression.

**Table 1:** Primer sequences.

Gene	Forward primer	Reverse primer
*18s* (28)	CTCAACACGGGAAACCTCAC	CGCTCCACCAACTAAGAACG
*Mmp2* (In-house)	TGGCAGTGCAATACCTGAACAC	AGTTGTAGTTGGCCACATCTGG
*Mmp12* (29)	CTGCTCCCATGAATGACAGTG	AGTTGCTTCTAGCCCAAAGAAC
*Csf3* (30)	ATGGCTCAACTTTCTGCCCA	CTGACAGTGACCAGGGGAAC
*Cxcl2* (31)	AGACAGAAGTCATAGCCACTCTCAAG	CCTCCTTTCCAGGTCAGTTAGC
*Il17a* (In-house)	TCTGTGTCTCTGATGCTGTTGC	GAGGTTGACCTTCACATTCTGG
*Ccl2* (32)	GTTGGCTCAGCCAGATGCA	AGCCTACTCATTGGGATCATCTTG
*Il6* (Primer ID 13624310c1) (33, 34)	CTGCAAGAGACTTCCATCCAG	AGTGGTATAGACAGGTCTGTTGG
*Tnfa* (Primer ID 7305585a1) (33, 34)	CCCTCACACTCAGATCATCTTCT	GCTACGACGTGGGCTACAG
*Il23a* (32)	ATGCTGGATTGCAGAGCAGTA	ACGGGGCACATTATTTTTAGTCT
*Nox2* (35)	CCCTTTGGTACAGCCAGTGAAGAT	CAATCCCGGCTCCCACTAACATCA
*Nox4* (Primer ID 7657389a1) (33, 34)	GAAGGGGTTAAACACCTCTGC	ATGCTCTGCTTAAACACAATCCT
*Sftpa1 (Origene)*	ACCTGGATGAGGAGCTTCAGAC	CTGACTGCCCATTGGTGGAAAAG
*Sftpb (Origene)*	TGTCCTCCGATGTTCCACTGAG	AGCCTGTTCACTGGTGTTCCAG
*Sftpc* (36)	TGATGGAGAGTCCACCGGATTA	CCTACAATCACCACGACAACGA
*Sftpd (In-house)*	AGGTCCAGTTGGACCCAAAGG	CTGGTTTGCCTTGAGGTCCTATG
*Abca3* (36)	GAGGGTCGGTGCCAGCACAT	GTCGCCTGGCGTCAGCAGTT
*Ttf1* (36)	TCCAGCCTATCCCATCTGAACT	CAAGCGCATCTCACGTCTCA

### 2.9 Statistics

Data were analyzed by nonparametric Mann-Whitney *U* test using GraphPad Prism version 9.0.1 for Windows (San Diego, CA, USA). Differences induced by DSS within one genotype are indicated by asterisks. To show the effect of TCRδ-deficiency on DSS-induced colitis in C57BL/6 mice, differences are indicated by a bar. Data points are presented as median ± interquartile range (IQR) and a *P* value less than 0.05 was considered significant.

## 3 Results

### 3.1 Deficiency of γδ T cells leads to development of more severe DSS-induced colitis

**Figure 1:**
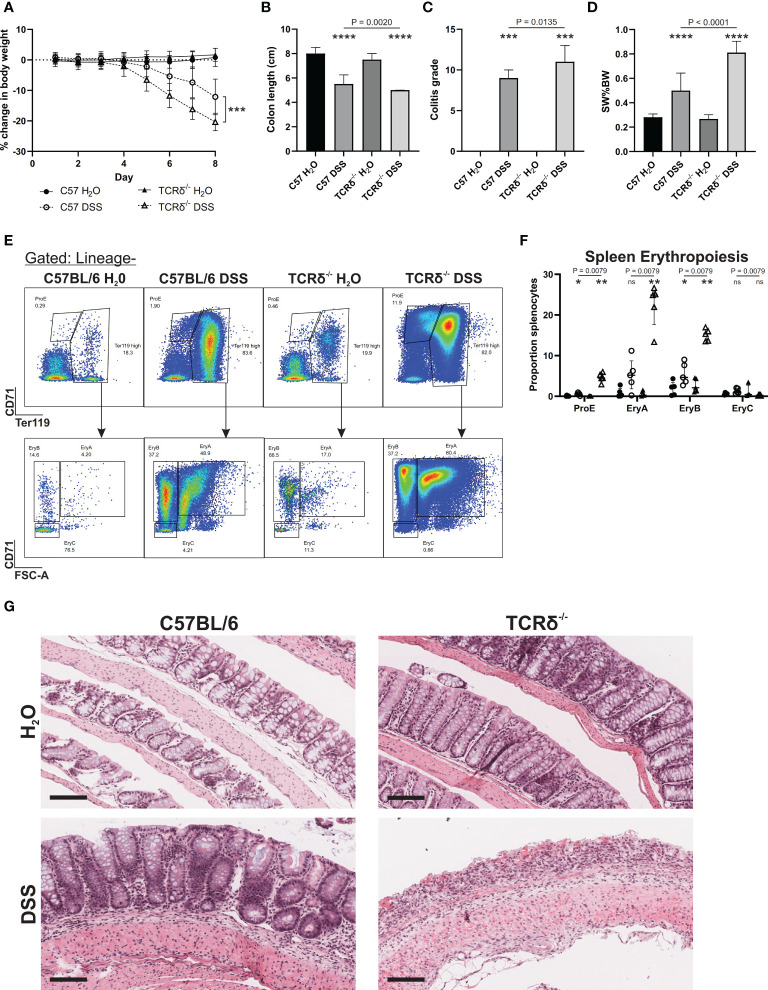
TCRδ^-/-^ mice exhibit an increased susceptibility to DSS-induced colitis. **(A)** Percentage change in body weight from day 0 of C57BL/6 (circle) or TCRδ^-/-^ mice (triangle) treated with either H_2_O (closed) or DSS (open). n = 18 – 30 mice per group. **(B)** Colon length (cm). n = 27 – 38 mice per group. **(C)** Histopathological colitis grade based on inflammation, epithelial injury, colitis activity, and lymphoid aggregates. n = 15 – 18 mice per group. **(D)** Spleen weight as a proportion of body weight (SW%BW). n = 18 – 26 mice per group. **(E)** Representative FACS plots showing erythropoiesis in spleen defined as ProE (CD71^high^Ter119^int^), large ‘EryA’ erythroblasts (CD71^high^Ter119^high^FSC-A^high^), smaller, more mature ‘EryB’ erythroblasts (CD71^high^Ter119^high^FSC-A^low^), and mature ‘EryC’ erythroblast (CD71^low^Ter119^high^FSC-A^low^) (37). Lineage = B220, CD11b CD62L, CD11b, Ly6G, Ly6C, F4/80. **(F)** Erythropoiesis as a proportion of splenocytes n = 5 mice per group. **(G)** Representative H&E-stained sections of Swiss rolls of colon from the indicated mice captured at 20x magnification. Scale bars = 100 μm. Representative of n = 15 – 18 mice per group. Data in B-E are presented as median ± IQR. ns, not significant; *p < 0.05; **p < 0.01; ***p < 0.001; ****p < 0.0001 by Mann-Whitney U test comparing water and DSS-treated mice of the same genotype.

TCRδ^-/-^ mice are more susceptible to intestinal inflammation induced by the gut toxin DSS than C57BL/6 mice (14, 17), which we confirmed by treating 12-week-old male mice (**Figure 1**). TCRδ^-/-^ mice exhibited greater weight loss following DSS treatment (**Figure 1A**), a greater reduction in colon length (**Figure 1B**), a more severe colitis grade (**Figure 1C**), enhanced splenomegaly (**Figure 1D**), and a marked increase in splenic erythropoiesis (**Figures 1E, F**) likely driven by severe rectal bleeding. Histopathology revealed pronounced epithelial erosion in DSS-treated TCRδ^-/-^ mice compared to DSS-treated C57BL/6 mice (**Figure 1G**).

### 3.2 Colitis induces neutrophilic inflammation in lung tissue

**Figure 2:**
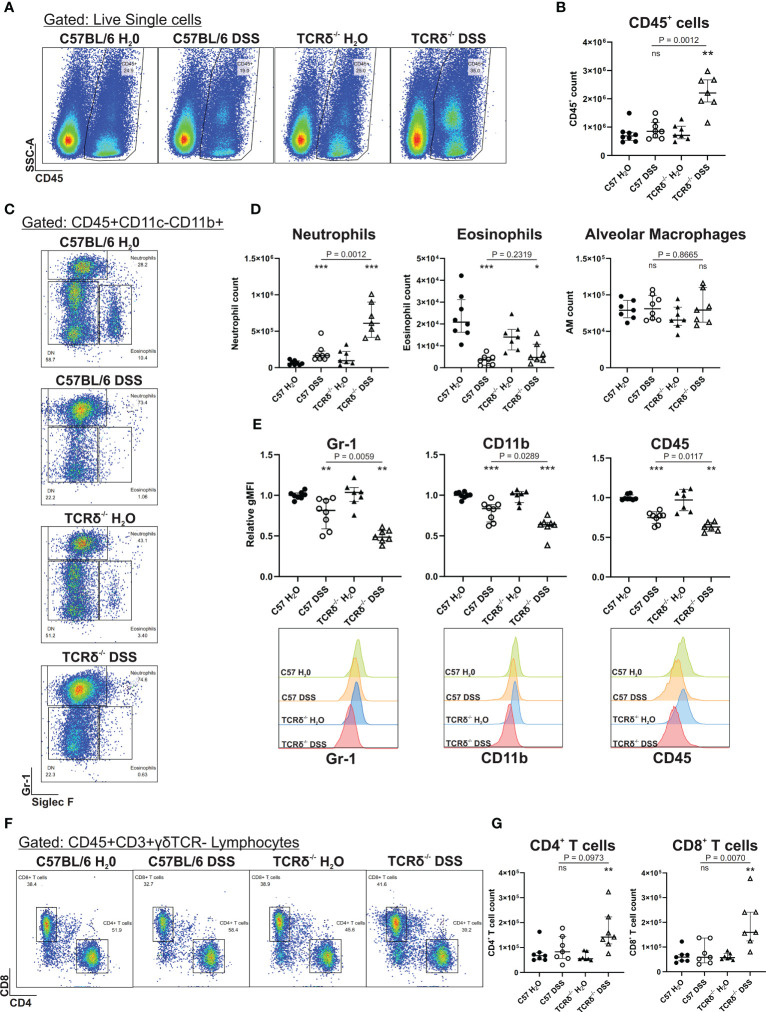
Induction of severe colitis in TCRδ^-/-^ mice induces neutrophilic lung inflammation. **(A)** Representative FACS plots showing frequencies of CD45^+^ cells vs side scatter (SSC-A) in digested whole lung tissue of C57BL/6 or TCRδ^-/-^ mice treated with either H_2_O or DSS. **(B)** Total number of CD45^+^ cells in whole lung tissue as determined by cell counts and flow cytometry from C57BL/6 (circle) or TCRδ^-/-^ mice (triangle) treated with either H_2_O (closed) or DSS (open). n = 7 – 8 mice per group. **(C)** Representative FACS plots depicting frequencies of neutrophils (CD45^+^CD11c^-^CD11b^+^SiglecF^-^Gr-1^+^) and eosinophils (CD45^+^CD11c^-^CD11b^+^SiglecF^+^Gr-1^-^) gated on CD45^+^CD11c^-^CD11b^+^ cells in whole lung. **(D)** Total number of neutrophils, eosinophils, and alveolar macrophages (CD45^+^CD11c^+^SiglecF^+^) in whole lung tissue derived by flow cytometry and cell counting. n = 7 – 8 mice per group. **(E)** Geometric mean fluorescence intensity (gMFI) of expression of Gr-1, CD11b, and CD45, and histogram of Gr-1 on lung neutrophils relative to C57BL/6 H_2_O. n = 7 – 8 mice per group. **(F)** Representative FACS plots depicting frequencies of CD4^+^ and CD8^+^ T cells gated on CD45^+^CD3^+^γδTCR^-^ lymphocytes in whole lung. **(G)** Total number of CD4^+^ and CD8^+^ T cells in whole lung tissue derived by flow cytometry and cell counting. n = 7 – 8 mice per group. Data is presented as median ± IQR. ns, not significant; *p < 0.05; **p < 0.01; ***p < 0.001 by Mann-Whitney U test comparing water and DSS-treated mice of the same genotype.

To determine whether increased severity of colitis may promote lung inflammation *via* the gut-lung axis in TCRδ^-/-^ mice, we digested whole lung tissue that had been previously lavaged. Flow cytometry analyses revealed that there was an increase in CD45^+^ leukocytes in the lung parenchyma of DSS-treated TCRδ^-/-^ mice, which was not seen in DSS-treated C57BL/6 mice (**Figures 2A, B**). Further assessment of the pulmonary CD45^+^ compartment, we observed a significant increase in neutrophils in DSS-treated C57BL/6 and TCRδ^-/-^ mice, as was observed in C57BL/6 mice previously (13), although numbers were significantly greater in TCRδ^-/-^ mice (**Figures 2C, D**). The neutrophils in both strains of DSS-treated mice exhibited an altered cell surface phenotype with reduced expression of Gr-1, CD11b and CD45, which was more greatly altered in the TCRδ^-/-^ mice (**Figure 2E**). Interestingly, there was a significant decrease in absolute numbers of eosinophils in the lungs of DSS-treated mice, while numbers of alveolar macrophages were unaffected (**Figures 2C, D**). We next assessed the lymphocyte compartment to determine if there were additional immune cell changes and found a slight but significant increase in CD4^+^ and CD8^+^ T cells in the lungs of DSS-treated TCRδ^-/-^ mice but not DSS-treated C57BL/6 mice (**Figures 2F, G**). Altogether, these data indicate that there is increased inflammation in the lungs of TCRδ^-/-^ mice following colitis characterized by a minor increase in T cells and a substantial influx of neutrophils.

### 3.3 Colitis-induced lung inflammation in wild type mice does not drive changes to γδ T cell numbers or activation marker expression

Previous studies have reported that TCRδ-deficient mice have increased susceptibility to lung infection, manifesting as augmented lung inflammation (18–20). To determine whether the protection from lung inflammation in C57BL/6 mice with colitis was due to the presence of γδ T cells the lung, we next assessed whether their numbers in different tissues changed in response to DSS. There was no significant difference in numbers of γδ T cells in blood, spleen, colon, or lung between mice with and without colitis (**Supplementary Figure 2A**). Furthermore, there was no significant change in activation of γδ T cells as measured by expression of CD62L (**Supplementary Figure 2B**). This suggests that protection from colitis-induced lung inflammation in C57BL/6 mice was not due to changes in γδ T cell numbers or activation, either systemically or in the lungs.

### 3.4 Neutrophils express an activated phenotype in TCRδ-deficient mice with colitis

**Figure 3:**
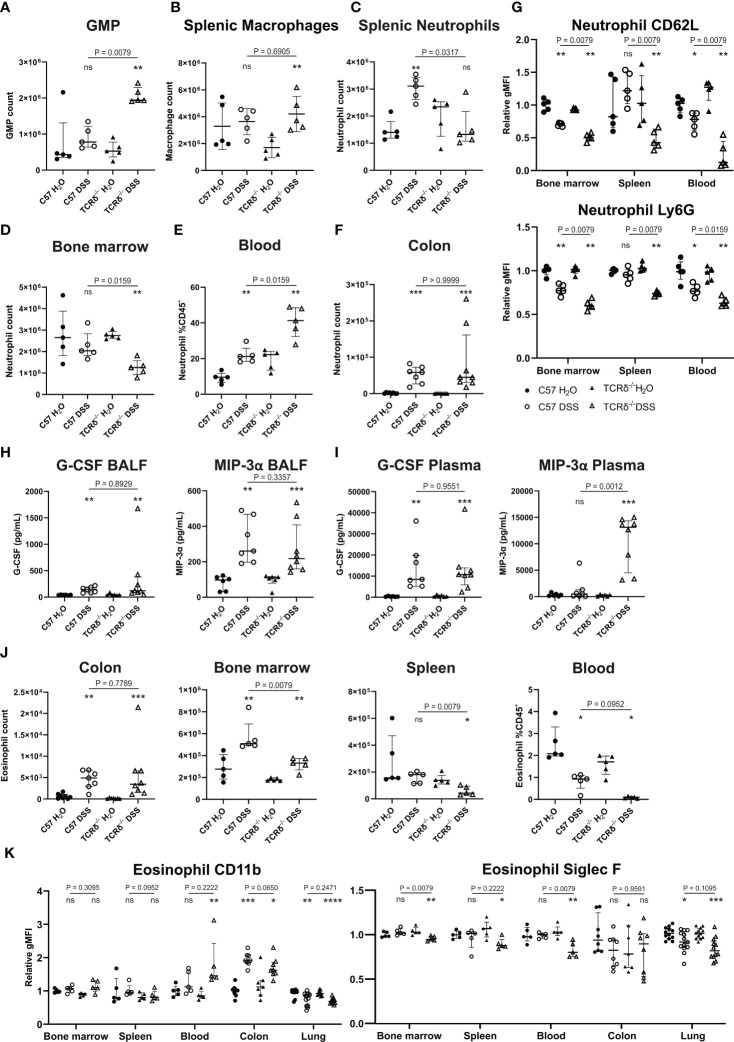
Colitis in TCRδ^-/-^ mice coincides with increases in neutrophil and neutrophil activation. **(A)** Total number of granulocyte/monocyte progenitors (GMPs) (CD45^+^Lin^-^Sca-1^-^cKit^+^CD16/32^+^) in spleen as determined by cell counts and flow cytometry from C57BL/6 (circle) or TCRδ^-/-^ mice (triangle) treated with either H_2_O (closed) or DSS (open). n = 5 mice per group. **(B)** Total number of macrophages (CD45^+^CD11b^-^CD11c^-^F4/80^+^) in the spleen as determined by cell counts and flow cytometry. n = 5 mice per group. **(C)** Total number of neutrophils (CD45^+^CD11b^+^Ly6G^+^) in spleen as determined by cell counts and flow cytometry. n = 5 – 8 mice per group. **(D)** Total number of neutrophils (CD45^+^CD11b^+^Ly6G^+^) in bone marrow determined by cell counts and flow cytometry. n = 5 – 8 mice per group. **(E)** Proportion of neutrophils (CD45^+^CD11b^+^Ly6G^+^) in blood as determined by flow cytometry. n = 5 mice per group. **(F)** Total number of neutrophils (CD45^+^CD11b^+^Ly6G^+^) in colon as determined by cell counts and flow cytometry. n = 5 – 8 mice per group. **(G)** Geometric mean fluorescence intensity (gMFI) of CD62L and Ly6G on neutrophils relative to C57BL/6 water controls in bone marrow, spleen, and blood. n = 5 mice per group. **(H)** Cytokine concentrations (pg/mL) of G-CSF and MIP-3α in the BALF. n = 6 – 8 mice per group. **(I)** Cytokine concentrations (pg/mL) of G-CSF and MIP-3α in plasma. n = 6 – 8 mice per group. **(J)** Total number of eosinophils in colon, bone marrow, and spleen, and proportions of eosinophils in blood as determined by cell counts and flow cytometry. n = 5 – 8 mice per group. **(K)** gMFI of CD11b and Siglec F expression on eosinophils relative to C57BL/6 water controls in bone marrow, spleen, blood, colon, and lung. n = 4 – 12 mice per group. Data in A-G is presented as median ± IQR. ns, not significant; *p < 0.05; **p < 0.01; ***p < 0.001; ****p < 0.0001 by Mann-Whitney U test comparing water and DSS-treated mice of the same genotype.

Since colitis induction drove a significant increase in neutrophils in the lungs of TCRδ^-/-^ mice, we next examined the myeloid compartment more carefully. Granulocyte/macrophage progenitors were increased in the spleen of TCRδ-deficient mice with colitis indicating an increase in extramedullary hematopoiesis (**Figure 3A**). This was associated primarily with an increase in splenic macrophages (**Figure 3B**). In C57BL/6 mice, DSS did not alter neutrophil numbers in the bone marrow, but it drove their expansion in the spleen and increased presence in the circulation (**Figures 3C–E**). Mature neutrophils were similarly expanded in the colon of both C57BL/6 and TCRδ^-/-^ mice treated with DSS (**Figure 3F**). In contrast, DSS induction of colitis in TCRδ^-/-^ mice led to a reduction in bone marrow neutrophils, no change in numbers in spleen but an increase in those in the circulation (**Figures 3C**–E). In mice with colitis, neutrophils had decreased CD62L and Ly6G expression in bone marrow and blood indicative of activation; however, this effect was significantly greater in TCRδ^-/-^ mice with colitis (**Figure 3G**). Reflective of the increase in neutrophils, there was increased granulocyte colony-stimulating factor (G-CSF) and MIP-3α (CCL20) in both the BALF and plasma of mice with DSS colitis (**Figures 3H, I**). Other cytokines assessed were for the most part unchanged (**Supplementary Figure 3**) other than IL-6, IL-22, and KC (CXCL1) which were likewise increased in plasma of both C57BL/6 and TCRδ^-/-^ mice with DSS colitis indicative of systemic inflammation (**Supplementary Figures 3C, D**).

Since we had observed a significant decrease in eosinophils in the lungs of TCRδ^-/-^ mice following colitis induction, we then further examined this cell subset. As has been previously observed, mice with DSS-induced colitis, regardless of genotype, had an increase in eosinophils in both the colon and the bone marrow (**Figure 3J**) (38). However, numbers of eosinophils were decreased in the spleen of TCRδ^-/-^ mice with colitis, and in the blood of both C57BL/6 and TCRδ^-/-^ mice harboring colitis (**Figure 3J**). Furthermore, evidence of eosinophil activation in blood and colon of TCRδ^-/-^ mice with colitis and colon of C57BL/6 with colitis was indicated by increased CD11b expression, although this coincided with decreased Siglec F expression (**Figure 3K**). These data show that induction of colitis drives an expansion of activated neutrophils, which can infiltrate the lungs of TCRδ-deficient mice to a greater degree. Colitis also induces an eosinophilia, although these cells target the colon and not the lung.

### 3.5 Lung damage is associated with severe DSS-induced colitis in mice lacking γδ T cells

**Figure 4:**
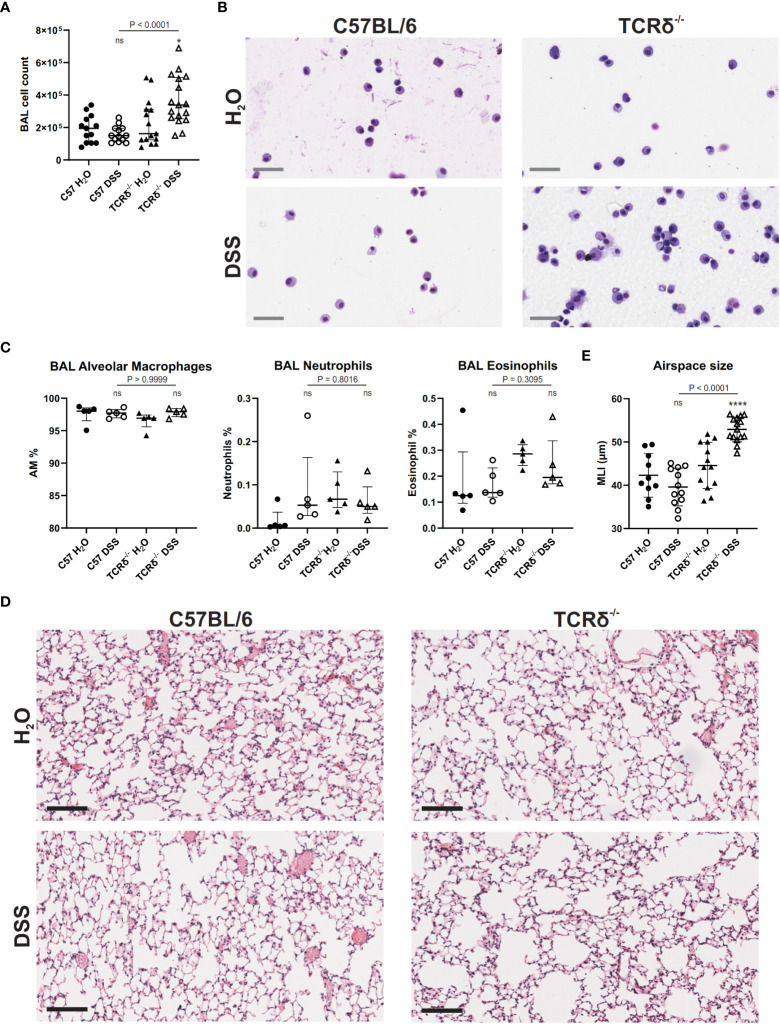
DSS-induced colitis in TCRδ^-/-^ mice promotes lung disease. **(A)** Total BAL cell counts from C57BL/6 (circle) or TCRδ^-/-^ mice (triangle) treated with either H_2_O (closed) or DSS (open). n = 13 – 17 mice per group. **(B)** Representative cytospins of BAL cells of the indicated mice stained with Hemacolor^®^ captured at 40x magnification. Scale bar = 50 μm. **(C)** Proportion of alveolar macrophages (CD45^+^CD11c^+^SiglecF^+^), neutrophils (CD45^+^CD11b^+^Ly6G^+^), and eosinophils (CD45^+^CD11b^+^SiglecF^+^) in BAL as determined by flow cytometry. n = 5 mice per group. **(D)** H&E-stained sections of inflation-fixed lungs of the indicated mice captured at 20x magnification. Scale bars = 100 μm. **(E)** Alveolar airspace size defined as mean linear intercept (MLI) in μm. n = 10 – 15 mice per group. Data is presented as median ± IQR. ns, not significant; *p < 0.05; ****p < 0.0001 by Mann-Whitney U test comparing water and DSS-treated mice of the same genotype **(A, D)**.

Since DSS treatment drove inflammation in the lung tissue, we next assessed the BAL to determine whether inflammation extended to entry of leukocytes into the airspaces. BAL cell quantification revealed no significant increase in leukocytes in the airspaces of DSS-treated C57BL/6 mice compared to water controls (**Figure 4A**) and cytospin analyses showed no change in cell morphology (**Figure 4B**). However, DSS treatment of TCRδ^-/-^ mice led to a significant increase in leukocytes in the airspaces (**Figure 4A**) and cytospin analyses suggested the presence of activated alveolar macrophages with occasional multinucleate giant cells, neutrophils, and eosinophils (**Figure 4B**), however there was no significant difference in proportions of alveolar macrophages, neutrophils, or eosinophils between groups (**Figure 4C**). This indicates that inflammation in the lung parenchyma progresses into the airspaces in the more susceptible model of colitis.

To assess whether increased inflammation in the lung tissue and airspaces of DSS-treated TCRδ^-/-^ mice promoted lung damage, histopathological analyses were performed. MLI measurements showed that TCRδ^-/-^ mice with colitis had larger airspaces when compared to TCRδ^-/-^ mice without colitis (**Figures 4D, E**). This was not observed in C57BL/6 mice with colitis, suggesting a loss of structural integrity in the TCRδ^-/-^ mice ordinarily provided by lung-resident γδ T cells during inflammation (**Figures 4D, E**). Together, these data demonstrate that mice lacking γδ T cells develop acute lung inflammation in parallel with more severe DSS-induced colitis. This inflammation is characterized by airspace enlargement as well as an increase in immune cell infiltration and expansion in the airspaces.

### 3.6 Increased pulmonary immune cell recruitment and activation following colitis

**Figure 5:**
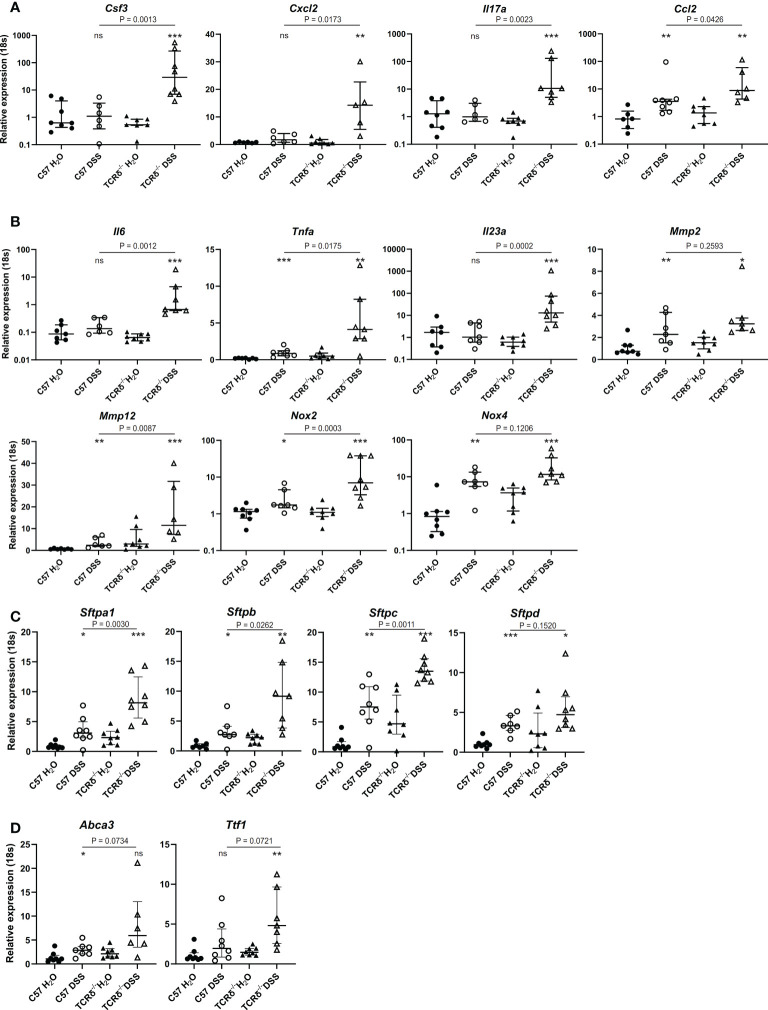
Increased expression of genes encoding cytokines, chemokines, lung structural elements and barrier function in lungs of mice with colitis. **(A–D)** Relative expression to *18S* in C57BL/6 H_2_O control mice of indicated genes in whole lung tissue from C57BL/6 (circle) or TCRδ^-/-^ mice (triangle) treated with either H_2_O (closed) or DSS (open). n = 6 – 8 mice per group. Data is presented as median ± IQR. ns = not significant; *p < 0.05; **p < 0.01; ***p < 0.001 by Mann-Whitney U test comparing water and DSS-treated mice of the same genotype.

To define possible mechanisms underlying lung damage in DSS-treated TCRδ^-/-^ mice, we analyzed the expression of genes encoding cytokines and chemokines known to contribute to predominately neutrophilic lung inflammation including *Csf3*, *Cxcl2*, *Il17a*, and the monocyte chemoattractant *Ccl2* (**Figure 5A**). TCRδ^-/-^ mice with colitis had significantly increased expression of these four genes, while only *Ccl2* was significantly increased in C57BL/6 mice with colitis albeit to a lesser degree than TCRδ^-/-^ mice (**Figure 5A**). Together, these data demonstrate that there is active recruitment of monocytes to the lungs of mice with DSS-induced colitis and neutrophils to the lungs of TCRδ^-/-^ mice harboring gut inflammation, which likely contribute to the associated lung damage.

Next, we assessed gene expression of cytokines produced by monocytes and macrophages that are involved in inflammation and lung tissue destruction. Elevated expression of *Il6*, *Tnfa*, and *Il23a* genes in whole lung tissue was seen in TCRδ^-/-^ mice with colitis, with only the *Tnfa* gene being mildly elevated in the corresponding C57BL/6 mice (**Figure 5B**). The gene encoding matrix metalloproteinase *Mmp2*, which is expressed by fibroblasts and endothelial cells in the lung but not immune cells, was similarly increased in whole lung tissue by DSS treatment of C57BL/6 and TCRδ^-/-^ mice, while *Mmp12* gene expression which is predominately expressed by macrophages, was mildly increased in C57BL/6 mice harboring colitis but markedly increased in whole lung tissue of TCRδ^-/-^ mice with colitis (**Figure 5B**). Similarly, gene expression of *Nox2* which is highly expressed in macrophages and to a lesser extent in neutrophils, was markedly increased in DSS-treated TCRδ^-/-^ mice compared to C57BL/6 mice (**Figure 5B**), while *Nox4*, which is expressed in fibroblasts but not immune cells was similarly upregulated in both C57BL/6 and TCRδ^-/-^ mice following DSS treatment. Taken together, these data demonstrate greater immune cell recruitment and activation to the lungs of TCRδ^-/-^ mice with colitis correlating with lung damage.

### 3.7 Changes in genes implicated in lung structure and barrier function in the lungs of mice with colitis

To determine whether inflammation in the lung following colitis affected expression of genes involved in lung structure, further gene profiling studies were done. Genes encoding surfactant proteins (SP), which are an important component of the lung barrier and essential to facilitate breathing (39), were amplified in the lungs of mice with DSS-induced colitis, with a greater increase observed in TCRδ-deficient mice (**Figure 5C**). Additionally, there was increased gene expression of *Ttf1* and *Abca3* which are known to regulate SP homeostasis (**Figure 5D**). These data suggest that in addition to inflammatory and structural changes in the lungs, colitis induces functional changes through induction of SP gene expression.

## 4 Discussion

IBD patients are more susceptible to chronic inflammatory lung diseases including COPD and asthma (2–5). Nevertheless, IBD patients harboring respiratory comorbidities represent an overlooked patient population from whom we can attain mechanistic insight into the gut-lung axis in the context of chronic inflammation. Previous studies assessing interactions between IBD and lung inflammation have been centered on epidemiological determinants or focused on the contribution of the microbiota (10). To investigate the role of the immune system in the gut-lung axis, we induced colitis in susceptible TCRδ-deficient mice and assessed the lungs for inflammation and pathology, providing a new model for evaluating the gut-lung axis in colitis.

Induction of colitis with the gut toxin DSS has been reported to induce neutrophilia in the lung parenchyma and an increase in leukocytes infiltrating the airways which is driven by an increase in circulating IL-6 (13). However, this previous study utilized C57BL/6 mice, which represent a milder model of lung effects in IBD compared to TCRδ^-/-^ mice reported here. In addition, we identified some differences between the response of C57BL/6 mice to DSS between the previous study (13) and our study; notably in our study, C57BL/6 mice with colitis did not have higher BAL cell counts. However, this is not necessarily surprising as it has been reported that different specific-pathogen free environments can result in large effects on inflammation in DSS-induced colitis (40). Furthermore, a recent study comparing genetically identical mice from the one facility found that variability in disease can be linked to variability in the microbiota (41), and germ-free mice are protected from inflammation with DSS colitis, although exhibit more susceptibility to epithelial injury (42). Similarly, microbiota, and thus housing, can also influence lung inflammation and pathology, with germ-free mice being protected from idiopathic pulmonary fibrosis (43). We observed elevated expression of *Il6* in whole lung tissue of TCRδ^-/-^ mice with colitis. IL-6 is constitutively expressed by the pulmonary epithelium in mice and this is elevated when these cells are activated, such as with allergen exposure (44). Additionally, macrophages and neutrophils can produce IL-6 in the inflamed lung (44). Thus, there is the potential for the expanding population of activated neutrophils and macrophages in the lungs of TCRδ^-/-^ mice after DSS-induced colitis to express and secrete IL-6 and elicit further immune responses. It would be interesting in the TCRδ^-/-^ model to assess whether this is tied to augmented bacterial load in the lung and whether it is to a greater extent than that observed in the less severe C57BL/6 DSS model.

The observed lung inflammation and pathology in TCRδ^-/-^ mice was unlikely to be due directly to deficiency in γδ T cells since there was no observed increase in γδ T cell activation or numbers in the lungs or systemically in C57BL/6 mice with colitis. This suggests that factors external to γδ T cell deficiency are driving lung inflammation in response to gut injury. In this model, inflammation in the lung is primarily characterized by neutrophil infiltration, driven by increased granulopoiesis in the spleen to expand circulating neutrophils, which infiltrate both the colon and the lung. This may indicate a spillover effect, where neutrophils produced to respond to the gut insult also infiltrate the lungs and consequently promote inflammation and lung damage. Interestingly, it has been reported that cigarette smoke-exposed mice exhibit an increase in neutrophils, amongst other myeloid cells in the small intestine and colon, suggesting lung to gut crosstalk (45). However, this study employed a whole-body cigarette smoke exposure model and smoke particulates may have induced gut inflammation *via* the oral route. Surprisingly, we also observed an increase in *Il17a* expression in the lungs of TCRδ^-/-^ mice with colitis. Due to γδ T cells being such a prominent source of IL-17A at mucosal sites such as the lung this was unexpected and suggests an alternative source of IL-17A in the inflamed lung (17, 46). This may well be neutrophils as they have been reported to be a source of IL-17A in certain inflammatory conditions (47–49). In addition to increased G-CSF and MIP-3α in the lungs of TCRδ^-/-^ mice with colitis, IL-17A is likely contributing to increased neutrophil recruitment to the lungs as has been observed in other models of lung inflammation (50). Thus, in addition to the proposed spillover effect, there is also active recruitment of neutrophils to the lung suggesting that factors within the lung itself may also drive inflammation during colitis.

Enlargement of alveolar airspaces following acute colitis coincided with increased expression of the genes encoding the elastolytic enzymes *Mmp12* and *Mmp2*, providing an explanation for the observed destruction of tissue (51). Additionally, we observed an increase in expression of *Nox2* and *Ccl2* suggesting macrophages are actively involved in damaging the lungs of TCRδ^-/-^ mice with colitis. Furthermore, colitis was associated with a decrease in lung eosinophils, which can be involved in tissue homeostasis and repair (52, 53), in addition to more destructive responses such as allergy and asthma (54). This is despite increased eosinophil production in the bone marrow and recruitment to the colon, indicating that colitis results in the specific recruitment of eosinophils to the gut, suggesting that the signals generated by the different tissues during inflammation and damage are distinct.

Pulmonary surfactant is well recognized for its role in increasing lung compliance to facilitate breathing (39). Additionally, SP-A and SP-D play critical antimicrobial roles essential for host defense (39, 55). These functions can be fully appreciated in either the absence of SP or when it is in excess, which result in changes in surface tension in the lung and thus altered lung function (55). Here we show that damage to the gut with DSS induces increased pulmonary expression of all four SPs genes, which, except for SP-D, were more significantly augmented in the lungs of TCRδ^-/-^ mice. Gene expression of the regulatory proteins *Abca3* and *Ttf1* were also increased and there were trending differences between C57BL/6 and TCRδ^-/-^ mice with colitis. SP are known to increase with pulmonary infection (56) and are induced by KGF signaling on alveolar type II cells (57). This suggests that severe colitis may promote antimicrobial inflammatory responses in the lungs.

There are limitations to our study. We show increased neutrophil numbers in the lung tissue based on flow cytometric analysis, however, do not describe neutrophil location within the lung or neutrophil function. Future studies are required investigate where in the lungs neutrophils are migrating and what cells they may be interaction with. Furthermore, neutrophil extracellular traps (NETs) have been associated with pulmonary diseases including COPD and asthma (58) and may also be worth investigating. It would similarly be interesting to investigate neutrophil depletion in future studies however G-CSFR^-/-^ mice develop a severe DSS-induced colitis (59) that suggests targeting neutrophils directly may have detrimental effects in the colon in the TCRδ^-/-^ mice which are already more susceptible to DSS-induced colitis (14, 16). Conversely, anti-IL-6 could be trialed in this model having shown some efficacy in C57BL/6 mice (13).

Here in this study, we assessed the immunological and histopathological changes that can be induced in the lungs in the presence of severe colitis to increase our understanding of the gut-lung axis. Such studies are important to aid in our understanding of lung involvement in IBD patients. Our data suggests that inflammation in the gut promotes an expansion of circulating innate immune cells that are specifically recruited to the lungs. In the presence of severe colitis, this drives pathophysiological changes within the lung *via* augmented recruitment and activation of neutrophils and macrophages, which promotes further inflammation and damage to the lung tissue. Additionally, increased SP production suggests augmentation of anti-microbial responses in the lungs which is likely a protective mechanism. This study has improved our understanding of how IBD promotes lung inflammation and the underlying mechanisms that should be targeted in the treatment or prevention of extraintestinal manifestations.

## Data availability statement

The raw data supporting the conclusions of this article will be made available by the authors, without undue reservation.

## Ethics statement

The animal study was reviewed and approved by Alfred Research Alliance Animal Ethics Committee.

## Author contributions

MH funded and supervised the research; AR, CO’B, and ET performed the research; AR analyzed the data; AR wrote the paper; and all other authors provided editorial comments. All authors contributed to the article and approved the submitted version.
