# Supplementary material for: Development of severe colitis is associated with lung inflammation and pathology
Source: Front Immunol. 2023 Mar 31;14:1125260. doi: 10.3389/fimmu.2023.1125260 (PMC10102339; doi:10.3389/fimmu.2023.1125260)
Supplement: Supplementary file 1 [file DataSheet_1.docx]

Supplementary Material

# Supplementary Figure


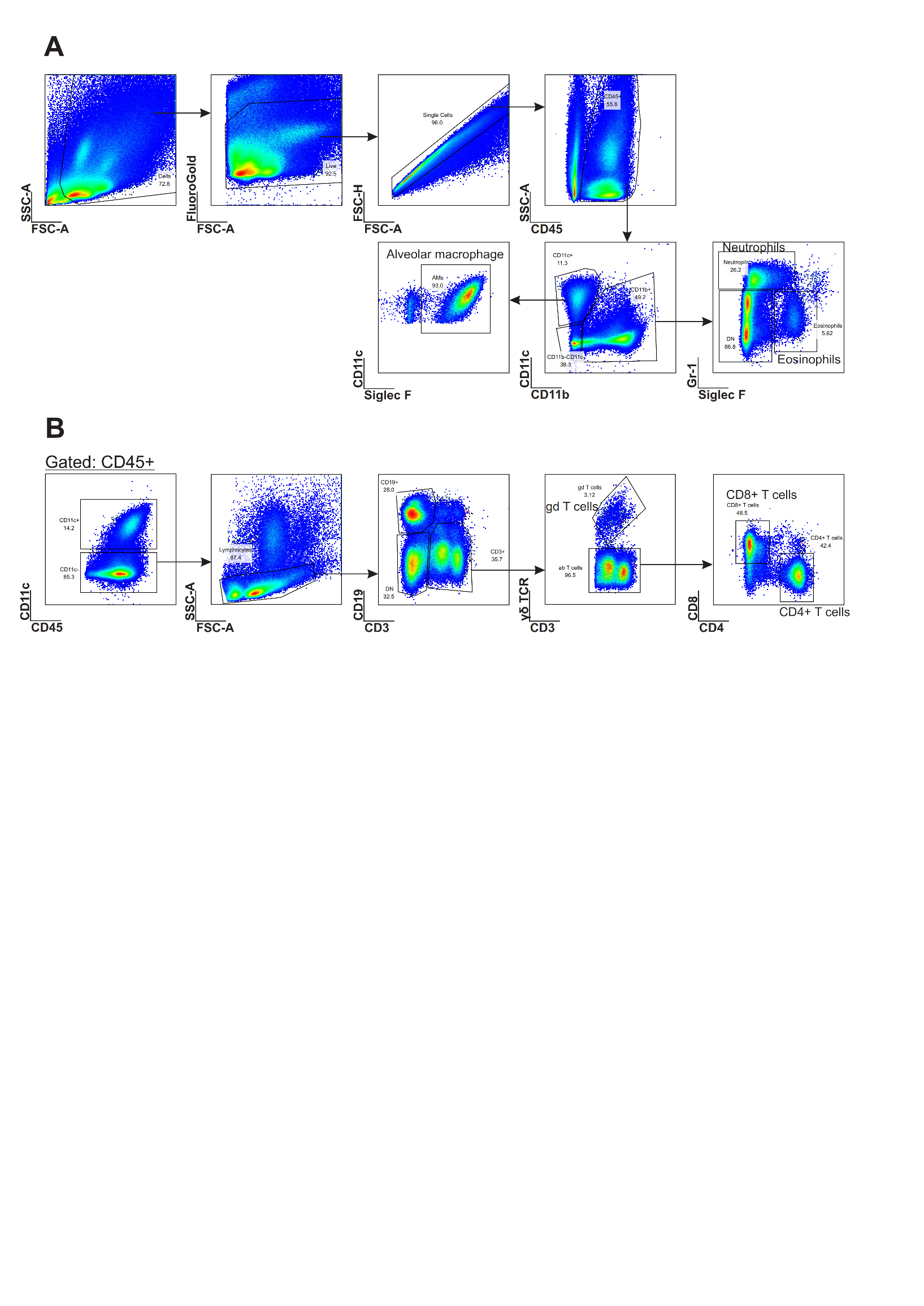
**Supplementary Figure 1. Gating strategy for flow cytometric analysis of lung.** Representative FACS plots from a C57BL/6 mouse on regular water outlining the gating strategy used to define key myeloid (**A**) and lymphocyte populations (**B**) in whole lung by flow cytometry.


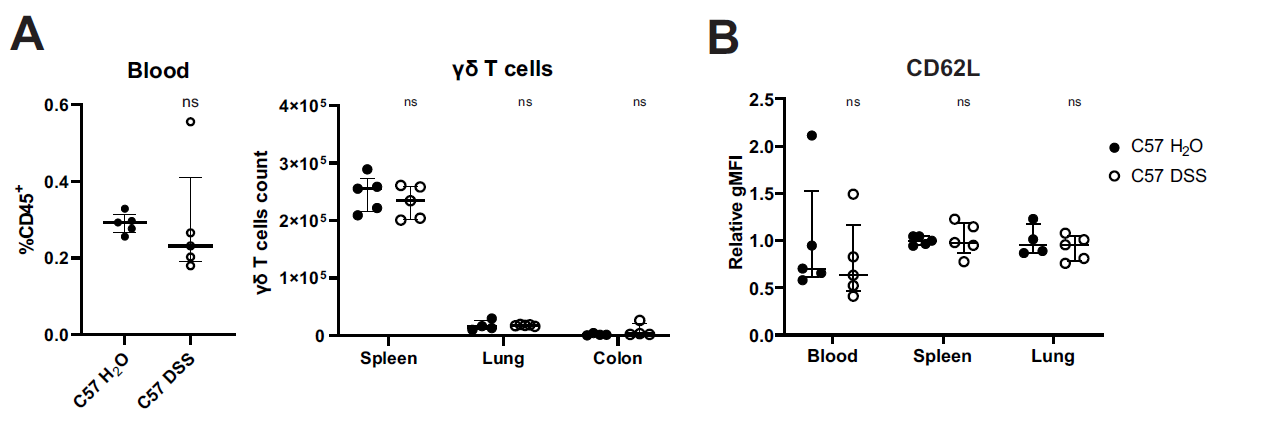


**Supplementary Figure 2. Colitis in C57BL/6 mice does not promote an increase in γδ T cells or γδ T cell activation.** **(A)** Proportions of γδ T cells in blood and total number of γδ T cells in spleen, lung, and colon as determined by cell counts and flow cytometry from C57BL/6 mice (circle) treated with either H_2_O (closed) or DSS (open). **(B)** gMFI of CD62L on γδ T cells relative to C57BL/6 water controls. Data represents at least n = 4 mice per group from 1 experiment. Data is presented as median ± IQR. ns = not significant by Mann-Whitney U test.

**
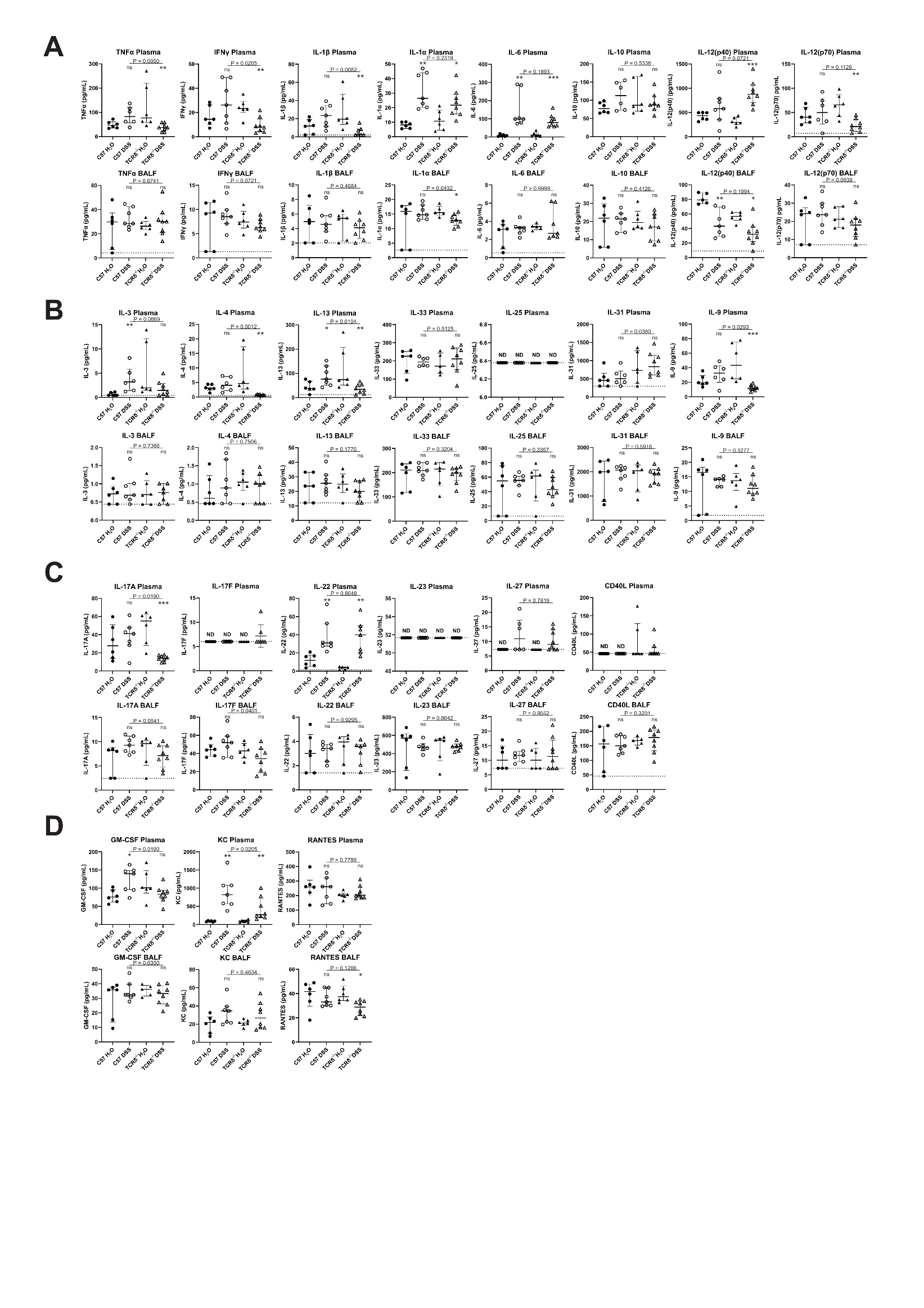
**

**Supplementary Figure 3. Cytokine measurements in plasma and BALF. A-D** Concentration (pg/mL) of indicated cytokines and chemokines in plasma and BALF from C57BL/6 (circle) or TCRδ^-/-^ mice (triangle) treated with either H_2_O (closed) or DSS (open) grouped by (**A**) type 1, (**B**) type 2, and (**C**) type 17 cytokines, and (**D**) chemokines. Dotted line indicates minimum level of detection. n = 6 – 8 mice per group. ns = not significant; * p < 0.05; ** p < 0.01; *** p < 0.001; **** p < 0.0001 by Mann-Whitney U test comparing water and DSS-treated mice of the same genotype.
